# Impact of culturally tailored shared medical appointments on diabetes self-care ability and knowledge in African Americans

**DOI:** 10.1017/S1463423623000166

**Published:** 2023-04-27

**Authors:** Adrienne L. Reddick, Deborah C. Gray

**Affiliations:** 1 Adjunct Professor, Old Dominion University, School of Nursing, Norfolk, VA, USA; 2 Associate Graduate Program Director, Clinical Associate Professor, Old Dominion University, School of Nursing, Norfolk, VA, USA

**Keywords:** African American, cultural tailoring, diabetes education, shared medical appointment, type 2 diabetes

## Abstract

**Background:**

Type 2 diabetes mellitus (T2DM) continues to disproportionately affect African Americans, significantly impacting morbidity and mortality. Research suggests that addressing barriers that stem from socioeconomic circumstances, systemic inequalities, biological factors, and cultural factors may positively influence biometric indicators of health and diabetes control.

**Objective:**

The aim of this study was to evaluate a diabetes shared medical appointment (SMA) model program that has been culturally tailored to address the unique social determinants of health barriers faced by an inner city African American population in Norfolk, Virginia.

**Methods:**

A pilot study using a within-group pretest–posttest design was conducted. Information was collected from self-report surveys that included the Modified Michigan Diabetes Knowledge Test, the Diabetes Self-Efficacy Scale, and researcher generated surveys before and after a single-session three-hour SMA program.

**Key Results:**

The program increased perceived diabetes self-care confidence and perceived overall diabetes knowledge levels. Increases in knowledge scores were seen but not statistically significant. Participants reported high levels of satisfaction with the program model.

**Discussion:**

Findings indicate that this model is an effective and engaging method of improving self-care ability and diabetes disease management knowledge among African Americans. Addressing unique circumstances and barriers experienced by this population may be more effective than usual traditional care approaches.

## Type 2 diabetes in African Americans

The incidence of type 2 diabetes mellitus (T2DM) in the United States continues to rise and African Americans remain disproportionately affected (Centers for Disease Control and Prevention, 2020). Black individuals are 60% more likely to be diagnosed with T2DM and twice as likely to die from complications when compared to their non-Hispanic White counterparts (Office of Minority Health, 2021). Non-Hispanic Black Americans are three times as likely to be hospitalized, over twice as likely to require lower limb amputation, and over three times as likely to be diagnosed with end-stage renal disease than White Americans as a direct result of T2DM (Office of Minority Health, 2021). Individuals with T2DM have health care costs that are an average of 2.3 times higher than those without T2DM, and they are more likely to experience increased disability, decreased productivity, and work absenteeism leading to perpetuation of socioeconomic instability (American Diabetes Association, 2018). Diabetes-related health disparities in the African American population are due to a complex interplay of biological and cultural factors, socioeconomic barriers, and systemic inequities (Bancks *et al*., 2017; Hicklin, 2018). These inequities often manifest as reduced access to resources, including healthy food and health care, neighborhood environments less conducive to promoting physical activity, economic instability, and a decreased level of trust in the health care system (Melillo, 2021).

## The shared medical appointment model

Shared medical appointments (SMAs) offer an innovative method of employing a voluntary group care model that provides education on disease prevention, disease management, and health promotion strategies to a greater number of recipients. The SMA model is designed to allow more time for patient education regarding disease prevention and management using a multidisciplinary interprofessional approach which may encourage the development of self-care skills (Wadsworth *et al*., 2019). While several different standardized diabetes SMA model programs have been developed, not all of them consider the unique characteristics of the population being served (Rashid *et al*., 2017; Hopkins DECIDE Program, 2019; Self-Management Resource Center, 2020). In general, the group-care model has shown positive effects on T2DM management as evidenced by improved biophysical health measures; however, the magnitude of these improvements show significant inter-study heterogeneity and may be greatly influenced by program design, provider experience, and population characteristics, with those more closely tailored to the population audience being more successful (Housden *et al*., 2013; Edelman *et al*., 2015; Watts *et al*., 2015; Wadsworth *et al*., 2019).

Optimal delivery models for SMAs vary depending on the population being served and remain understudied. Information available suggests that effective diabetes care and patient self-management often require extensive patient education and skill acquisition that may be enhanced when cultural preference and values are considered through cultural tailoring. Cultural tailoring that goes beyond simple language translation and includes content that addresses salient values, customs, dietary habits, community, and religion has been shown to increase the effectiveness of diabetes education programs within ethically, racially, and culturally diverse groups (Hu *et al*., 2016; Choi *et al*., 2017; Rosas *et al*., 2018). Within African American communities, group education and SMA programs that utilize culturally tailored methods have previously been shown to have positive influences on biometric markers and lifestyle changes among participants (Newby and Gray, 2016). Overall, there remains a lack of information on the effects of culturally tailored SMAs on patient knowledge of T2DM disease process and management, perception of self-care abilities, and satisfaction, particularly with regard to culturally tailored SMA models for African Americans (Hernandez *et al*., 2016; Drake, 2019).

## The culturally tailored diabetes SMA program

The Healthy Living Center is a 501(c)3 nonprofit organization that offers a unique culturally tailored diabetes SMA model program specifically adapted to for low-income, underserved, and/or medically vulnerable African Americans living in Norfolk, Virginia. The program is aimed at increasing health education and awareness using an interactive group model approach. Participants initially report to the affiliated clinic, Primary Care Specialists, where they have a one-on-one visit with a nurse practitioner (NP) or medical doctor (MD). At that time, participants have a diabetes-focused exam and are given the opportunity to discuss individual treatment plans, any concerns, and ask personal questions. Participants are provided with printouts of their most recent diabetes-related laboratory information and biometric data before being escorted to the Healthy Living Center, located in the same building.

The three-hour group education portion of the SMA takes place in a classroom and simulated kitchen setting with 8–12 participants. Instructors are community-based African American medical professionals from the local Primary Care Specialists clinic who are intimately familiar with the social determinants of health barriers among Norfolk’s inner-city African American community. Curriculum design was informed by these barriers which include a lack of access to affordable healthy food options, low health literacy, and difficulty effectively integrating diet and healthy lifestyle recommendations. The team of instructors includes a MD, NPs, and a pharmacist. The core curriculum follows the Association of Diabetes Care and Education Specialists (ADCES) 7 Self-Care Behaviors education format which incorporates the following elements: healthy coping; healthy eating; being active; diabetes monitoring; medication adherence; problem-solving; and reducing health risks (Association of Diabetes Care and Education Specialists, 2020). Teaching methods are aimed at acknowledging and addressing cultural norms and preferences of participants and include a combination of PowerPoint presentations, group discussions, demonstrations, and interactive activities to engage participants and to promote discussion and peer support. Personal laboratory information and biometric data printouts are referenced throughout the SMA to help participants understand how diabetes is clinically monitored and how to interpret their personal laboratory information with the goal of improving health literacy. Instructors used vernacular and a level of teaching appropriate to the population and incorporated culturally specific examples and hypothetical scenarios to enhance the relatability and impact of the lessons. A strong emphasis is placed on educational content pertaining to culturally preferred foods, paired with interactive experiential activities such as cooking demonstrations that feature healthier food choices and alternatives to common unhealthy means of preparing traditional African American dishes.

## Methods

A convenience sample of participants was recruited over a six-month period from individuals enrolled in a recurring single three-hour interactive culturally tailored diabetes SMA model group care and educational interactive learning session at the Healthy Living Center. The standardized SMA program is scheduled on the third Thursday of each month with a new cohort attending each session. Recruited participants (N = 37) included African Americans aged 33 to 83 years with a confirmed diagnosis of T2DM (Table 1). Individuals were excluded from the study if they were under 18 years of age or over 90 years of age, had not been diagnosed with T2DM, were not proficient in English, or did not identify as African American. The duration of the study was six months.

## Data collection

Information was collected from self-report surveys administered to participants before and after the single-session culturally tailored diabetes SMA model program. The Diabetes Self-Efficacy Scale (DSES) was used to evaluate pre- and post-intervention differences in perceived self-care ability. It is comprised of eight questions that are answered using a Likert scale designed to measure self-efficacy for common diabetes-related behavioral and medical management issues. The Modified Michigan Diabetes Knowledge Test (MMDKT) was used to evaluate pre- and post-knowledge levels of the T2DM disease process and management. The modified version used in this study is written in a true/false/don’t know format (Fitzgerald *et al*., 2016; Michigan diabetes Research Center, 2020). Ten of the original modified MMDKT questions were chosen and reviewed for appropriateness by two NPs and an MD familiar with the study population to create an abridged version of the MMDKT which was more appropriate for the health literacy levels of the study population. Participants self-rated their diabetes knowledge level before and after the SMA, as well as perceived improvement in diabetes knowledge after attending the SMA. All instruments were reviewed for face and content validity by a group of medical and diabetes experts familiar with the study target population and were pilot-tested with a small sample to determine ease of use and the clarity of instructions and questions.

## Results

The mean total DSES score before the intervention was 54.65 points (SD = 16.816), and the mean total score after the intervention was 68.53 (SD = 10.255) (Table 2). DSES total potential scores range from 8 to 80. This difference indicated that attending the culturally tailored diabetes SMA model program resulted in a statistically significant overall increase in participants’ perceived diabetes self-care confidence after attending the culturally tailored diabetes SMA model program (t = −5.173, *P* < 0.001) (Table 2). The Wilcoxon signed-rank test showed a statistically significant difference in participants’ self-reported comfort in checking blood sugar levels after participating in the intervention (Z = −2.333, *P* = 0.020).

**Table 1.** Participant demographic data

Variable	N	%	
Gender (N = 37)			
Female	26	70.3	
Male	10	27.0	
No answer provided	1	2.7	
Level of education (N = 37)			
Less than a high school diploma	2	5.4	
High school graduate	13	35.1	
Some college	14	37.8	
Associate degree	4	10.8	
Bachelor’s degree	4	10.8	
Graduate degree	0	0	
	Mean (SD)	Median	Range
Age (years) (N = 37)	58.43 (11.80)	59.0	33–83

**Table 2.** Differences in pre- versus post-intervention diabetes self-care confidence levels (DSES)

Question	Pre-intervention self-reported confidence level			Post-intervention self-reported confidence level				
	Mean	SD	N	Mean	SD	N	Paired t-test	p
How confident do you feel that you can eat meals every 4 to 5 h every day, including breakfast?	6.54	3.042	35	7.54	2.477	35	−1.839	0.075
How confident do you feel that you can follow your diet when you have to prepare or share food with other people who do not have diabetes?	6.63	2.840	35	7.91	1.869	35	−2.684	0.011
How confident do you feel that you can choose the appropriate foods to eat when you are hungry?	6.91	2.811	35	8.40	1.769	35	−2.835	0.008
How confident do you feel that you can exercise 15 to 30 min, 4 to 5 days a week?	6.63	2.941	35	8.20	1.967	35	−3.847	0.001
How confident do you feel that you can do something to prevent your blood sugar level from dropping when you exercise?	6.71	3.020	34	9.06	1.229	34	−4.522	<0.001
How confident do you feel that you know what to do when your blood sugar level goes higher or lower than it should be?	6.11	2.888	35	9.00	1.213	35	−6.155	<0.001
How confident do you feel that you can judge when the changes in your illness mean you should visit the doctor?	7.74	2.895	34	9.29	1.219	34	−3.287	0.002
How confident do you feel that you can control your diabetes so that it does not interfere with the things you want to do?	7.17	2.491	35	9.11	1.301	35	−4.648	<0.001
**Diabetes self-efficacy** **Scale score total**	54.65	16.816	34	68.53	10.255	34	−5.173	<0.001

Although there was an increase in mean total MMDKT scores after the intervention, the increase was not statistically significant (Table 3). However, individual question analysis revealed improvements in understanding of the following: impact of unsweetened fruit juice on blood glucose levels; recognizing signs of neuropathy; understanding organs affected by diabetes; the difference between foods high in protein versus carbohydrates; how diet soda differs from regular soda in terms of sugar content; the inevitability of disease progression; and how illness affects blood sugar levels (Table 3). Additionally, self-assessed perceived differences in overall diabetes knowledge levels were also evaluated and found to be significantly higher post SMA (Z = −3.314, *P* = 0.001).

**Table 3.** Difference in pre- versus post-intervention knowledge test questions (MMDKT)

Knowledge test question	Pre-intervention		Post-intervention		Change	McNemar test	
	% Correct	N	% Correct	N	%	p	N
Having regular check-ups with your doctor can help identify the early signs of diabetes complications	94.4	36	94.1	34	−0.3	1.000	33
Numbness and tingling may be symptoms of nerve disease	66.7	36	82.4	34	15.7	0.070	33
Infection is likely to cause an increase in blood sugar levels	69.4	36	76.5	34	7.1	0.250	33
A pound of chicken has more carbohydrates in it than a pound of potatoes	61.1	36	73.5	34	12.4	0.344	33
Lung problems are usually associated with having diabetes	41.7	36	55.9	34	14.2	0.607	33
A can of diet soda can be used for treating low blood glucose levels	36.1	36	47.1	34	11.0	0.581	33
Unsweetened fruit juice raises blood glucose levels	22.2	36	41.2	34	19.0	0.092	33
High blood glucose levels may be caused by too much insulin	47.2	36	38.2	34	−9.0	0.774	33
Attending your diabetes appointments stops you from getting diabetes complications	25.0	36	35.3	34	10.3	0.344	33
Glycosylated hemoglobin (HbA1c) is a test that measures your average blood glucose level in the past week	27.8	36	25.7	35	−2.1	1.000	34

	Mean		Mean		Paired t-test		
	% Correct	Score	% Correct	Score	*t*	*p*	
**Modified Michigan Diabetes Knowledge Test Score Total**	51.2	5.12	57.0	5.70	−1.316	0.197	

Participants used a Likert scale to rate satisfaction with the culturally tailored diabetes SMA model program on a scale of 1 (not at all satisfied) to 10 (extremely satisfied) upon completing the program. The mean satisfaction score was 9.82 (range 8–10; SD = 0.459).

## Discussion and implications

Innovative diabetes interventions are necessary to respond to the growing disparities in diabetes prevalence and outcomes among minority groups in the United States. The ongoing deep-rooted racial disparities seen in diabetes management and outcomes indicate that the current approach to diabetes care is not adequately tailored to meet the needs of the African American population. Designing diabetes education and self-management content and activities to better meet the requirements of specific patient populations may strengthen patients’ ability to assimilate self-care recommendations into their daily lives. Interactive culturally tailored models, such as this one, that are also led by African American clinicians, may be more influential in empowering African American individuals to set and achieve personal health goals by instilling confidence in self-care abilities. The SMA program examined here can be used as a model for adapting traditional diabetes care to develop engaging patient-centered programs that better serve culturally or ethnically diverse populations. This pilot study supports that this culturally tailored diabetes SMA model is a highly acceptable method of addressing diabetes care needs in African Americans, while significantly enhancing self-care confidence. While statistical significance in improved knowledge levels as measured by the MMDKT scores was not demonstrated, findings suggested both actual and perceived noteworthy knowledge acquisition from the SMA program which could prove to be clinically significant in terms of improved disease outcomes.

## Limitations

COVID-19 gathering restrictions significantly limited the number of patients that could enroll in the SMA or participate in the study, resulting in a small sample size (N = 37). Additionally, despite efforts to minimize effects, low literacy levels in the sample may have impacted the ability of some participants to fully understand the questions and provide accurate answers on the self-report surveys. Future studies with a larger sample and additional data collection methods may strengthen findings.

## Conclusion

We have outlined an innovative program that considers the multifactorial interplay of social determinants of health, institutional racism, biological differences, and widespread socioeconomic disparities unique to African Americans that shows improvement in perceived self-efficacy levels and self-rated knowledge levels, while simultaneously having high levels of participant satisfaction. The findings from this pilot study support future efforts to further explore the effectiveness of this culturally tailored model for improving diabetes knowledge and self-management ability among minority and underserved populations.
